# Comparing the nutritional composition of foods and beverages in the Canadian Nutrient File to a large representative database of Canadian prepackaged foods and beverages

**DOI:** 10.1371/journal.pone.0280028

**Published:** 2023-03-13

**Authors:** Jodi T. Bernstein, Anthea K. Christoforou, Nadia Flexner, Mary R. L’Abbe

**Affiliations:** Department of Nutritional Sciences, Temerty Faculty of Medicine, University of Toronto, Toronto, Ontario, Canada; University of Catania: Universita degli Studi di Catania, ITALY

## Abstract

**Background:**

Nutrient information used to code dietary intakes in the Canadian Community Health Survey (CCHS) may not be reflective of the current Canadian food supply and could result in inaccurate evaluations of nutrient exposures.

**Objective:**

To compare the nutritional compositions of foods in the CCHS 2015 *Food and Ingredient Details (FID)* file (n = 2,785) to a large representative Canadian database of branded food and beverage products (Food Label Information Program, FLIP) collected in 2017 (n = 20,625).

**Method:**

Food products in the FLIP database were matched to equivalent generic foods from the FID file to create new aggregate food profiles based on FLIP nutrient data. Mann Whitney U tests were used to compare nutrient compositions between the FID and FLIP food profiles.

**Results:**

In most food categories and nutrients there were no statistically significant differences between the FLIP and FID food profiles. Nutrients with the largest differences included: saturated fats (n = 9 of 21 categories), fiber (n = 7), cholesterol (n = 6), and total fats (n = 4). The *meats and alternatives* category had the most nutrients with significant differences.

**Conclusion:**

These results can be used to prioritize future updates and collections of food composition databases, while also providing insights for interpreting CCHS 2015 nutrient intakes.

## Introduction

The Canadian population-level national dietary survey, the Canadian Community Health Survey (CCHS) includes food and nutrition-related modules that provide detailed data on food consumption in Canada [1, 2]. Results from CCHS nutrition surveys have immense applications in nutrition and agricultural policy decisions, steer research activities examining nutrient disease relationships, influence the development of standardized nutrient reference amounts, and inform dietary programs and advice [3]. Food items in the most recent nutrition iteration of the CCHS, CCHS 2015, used a subset of the 2015 Canadian Nutrient File (CNF) to code food items reported in the survey’s 24-hour recall, these food items are found in the CCHS *Food and Ingredient Details* (FID) file. The CNF database is the standard reference food composition database for the Government of Canada. It includes information on the amounts of nutrients in the types of foods that are commonly consumed in Canada [4]. The CNF is composed of food profiles that are primarily generic representative composites (e.g. “*Bread*, *spelt*, *commercial*”). The CNF is used by a number of Government of Canada agencies including, Statistics Canada, Health Canada, Agriculture and Agri-Food Canada, and the Canadian Food Inspection Agency [4]. However, using the CNF to analyze the current food supply or Canadian intakes poses several challenges due to its lack of scheduled, comprehensive and systematic updating and the use of non-Canadian food composition data. Data in the CNF database was obtained largely from the National Nutrient Database for Standard Reference published by the United States Department of Agriculture, with the exception of foods known to be absent in the Canadian market [5]. Nutrient levels for items from the National Nutrient Database for Standard Reference were then modified to reflect regulatory standards for Canadian fortification levels [5]. The CNF was then further supplemented with Canadian-only foods and Canadian commodity data [5]. Furthermore, each edition of the CNF is updated for food categories that are determined to be of highest priority [5]. Since 2007, the CNF has been updated using the SNAP-CAN program, which outlines sampling and analysis protocols and aims to sample the top selling brands representing >85% of the consumer market for the priority foods identified [6]. However, comprehensive updates of all food profiles in the CNF are not done due to limited resources [5]. Thus, the CNF may not be reflective of the current Canadian food market. Inaccurate food composition data has the potential to lead to erroneous results in research, poor policy decisions, and misinformed food selection [3]. As such, timely, accurate, and geographically-specific data are required for analyzing intakes in the context of a rapidly changing food supply, particularly with the policy priorities focused on reducing levels of nutrients of public health concern [7–11]. The primary aim of this study was to compare the nutritional composition of the food profiles in the CCHS 2015 FID file, that is composed almost entirely of food items from the CNF 2015 database to equivalently matched products in a large representative database of prepackaged food and beverage products available in the Canadian food supply in 2017.

## Methods

This study is a cross-sectional analysis of two food composition databases, the CCHS 2015 FID file derived from the CNF and the University of Toronto’s Food Label Information Program (FLIP) 2017 database, which the latter contains comprehensive nutritional composition of prepackaged food and beverages available for sale in Canadian grocery stores.

### CCHS 2015 Food and Ingredients Detail (FID) file

The FID file contains the nutrient information for basic food recipes and ingredients (“food profiles”) (n = 2785). Each FID food profile has an ID number, nutrient composition, food group, in addition to other items of information (e.g. CCHS participant sample ID and recall number) not relevant to the analysis conducted in the current study [12]. To facilitate comparisons by food category, all items in the FID file were categorized into major food groups based using the existing taxonomy for Health Canada’s Table of Reference Amount major categories [13].

### Food Label Information Program (FLIP) 2017 database

The FLIP 2017 database is a food composition database that includes information on the nutritional composition, UPC, company, brand, price, ingredients, container size, store of collection, and sampling date for both national and private-label prepackaged foods and beverages (n = 17,629). Data for FLIP 2017 were collected in the Greater Toronto Area from the three largest grocery chains in Canada by market share (i.e. Loblaws, Sobeys, and Metro), representing approximately 65% of the grocery retail market share. Specific details on the FLIP database have been previously described [14]. Nutrient composition information for calories (kcal), total fat (g), saturated fats (g), trans fats (g), sodium (mg), cholesterol (mg), carbohydrates (g), sugars (g), fibre (g), and protein (g) as per the manufacturer stated serving size were obtained from the NFt then converted to standardized units (per 100g). Products in FLIP 2017 that were sold in their unprepared form (not ready-to-eat) were prepared according to manufacturer provided instructions, with the addition of water or other ingredients, when appropriate (n = 1271). The nutritional composition of the “prepared” version of such FLIP products was determined using ESHA Food Processing Software. Additionally, conversion factors were used for FLIP products to adjust for the volume change between the prepared and unprepared versions (e.g. pasta, rice, beans, doughs) (n = 1676). Furthermore, secondary preparations of FLIP products were also made when there were food profiles in the FID file that required the additional preparation for matching (process described below) (n = 49). This was only done if it did not conflict with the manufacturer provided instructions, for example, the preparation of chocolate milk with either whole or 2% milk when the manufacturer only specified that “milk” should be added. In total there were n = 20,625 FLIP products (both prepared and unprepared), available to be matched with an FID food profile.

### Matching FID file food profiles and FLIP products

Food and beverage products in FLIP 2017 were matched to FID food profiles as if they were being reported as consumed in a CCHS 24-hour recall. Details on the food and beverage coding process was obtained through discussions with staff at Health Canada to ensure the matching followed a similar process to coding of foods and beverages reported as consumed during the CCHS survey. Health Canada staff also shared their *Default List* for coding foods reported in CCHS 2015 24-hour recalls [15]. This list was used to help decision-making when the coding of reported foods and beverages was not immediately obvious. The *Default List* was not exhaustive and contained coding suggestions for only 835 food and beverage items. For each product in the FLIP database, the FID file was surveyed to identify a match using the decision-making steps outlined in **Table 1**, ranging from most to least objective. We attempted to match all FLIP products with an FID food profile, except when there was a better match with a recipe, found in the CCHS *Food Recipe Level* file (e.g. lasagna, prepared pudding mixes) (n = 4,429) or when the FLIP product was a combination of items that were assumed to have been easily reported as separate foods (e.g. hummus and cracker kit) (n = 60). *Food Recipe Level* items are composed of ingredients found in the FID file. Due to the nature of the FLIP database, the nutrition information from the Nutrition Facts table for several combination items could not be separated for each component. One researcher who is a Registered Dietitian, identified matches for all FLIP products by running through the steps outlined in Table 1. When the decision-making was considered subjective (matched at Step 5) or if the first researcher determined additional consensus was required (n = 968), another researcher who is a Nutritionist, provided input in the matching process until a consensus was reached by both researchers. Each FLIP product was matched to only one FID food profile unless there was an additional FID food profile for the same food in its prepared, cooked, or heated version. In this case, a FLIP product could be matched multiple times for different preparation methods.

**Table 1:** Step-by-step method for determining which FID food profile was most appropriate to match with each FLIP product and the number and percent (%) of foods matched at each step (n = 16,136) ^a^.

Step	Description	n (%)
1 –Exact Matches	FLIP product was matched to the *one and only* FID food profile name that accurately describes the FLIP product.	11,292 (70.0%)
2 –Default Matches	FLIP product was matched to the FID food profile suggested in Health Canada’s *Default List*.	709 (4.4%)
3 –Closest Matches	FLIP product was matched to the *one and only* FID food profile that is almost an exact match to the FLIP product.	1332 (8.3%)
	e.g. a FLIP product that is a hard candy sweetened with maltitol, matches to the FID food profile for “Candies, hard, sorbitol sweetened”.	
4 –Ingredient Match	FLIP product was matched to *one of several* potential FID food profiles based on the order of the FLIP product Ingredients List or the FLIP product description.	333 (2.1%)
	e.g. a FLIP product that is a blend olive and canola oils, with olive oil as the primary ingredient, matches to the FID food profile for “vegetable oil, olive”.	
5 –Judgement Match	FLIP product was matched to *one of several* potential FID food profiles based on the researchers’ determination of which would be the most similar in terms of nutrient profile and consumer use of the product.	1476 (9.1%)
6 –No match	FLIP product was not matched to an FID food profile if the researchers determined there was no appropriate match (e.g. FLIP product that is chia pudding) or if there was a difference in preparation methods (e.g. FLIP product that is unprepared noodle soup and the closest FID food profile is for the prepared version).	994 (6.2%)

^a^ In total, there were 20,625 FLIP products (in both prepared and unprepared forms) available to be matched, 4,429 were better matched to items in the CCHS *Food Recipe Level* file and 60 were identified as combination foods that would likely be reported separately, leaving 16,136 available to be matched to FID food profiles.

Abbreviations: FID = Food and Ingredients Details; FLIP = Food Label Information Program.

### Creation of aggregated nutritional information for FLIP products

**Fig 1:**
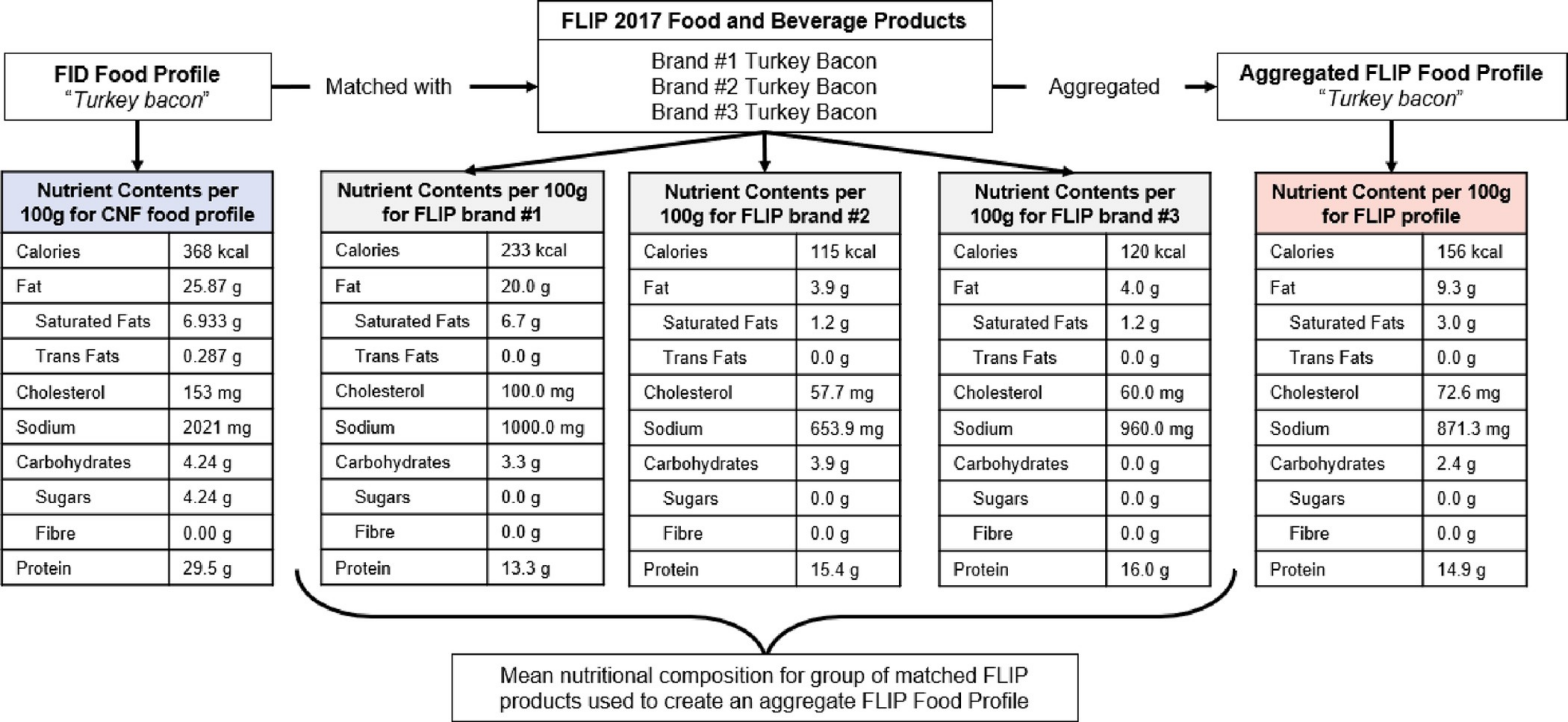
Creation of an aggregate nutritional composition (FLIP food profile) using the nutritional information for FLIP products matched to the same FID food profile. Abbreviations: FID = Food Ingredient Details; FLIP = Food Label Information Program database.

An aggregate of the nutritional information for FLIP products was created by determining the average nutrient information initially derived from the product Nutrition Facts table (calories (kcal), total fat (g), saturated fats (g), trans fats (g), sodium (mg), cholesterol (mg), carbohydrates (g), sugars (g), fibre (g), and protein (g) per 100g) for all FLIP products matched to a single FID food profile (see **Fig 1**). The result was the creation of a FLIP food profile that was used for comparisons with the FID food profiles.

### Statistical analysis

The number and proportion (%) of FID food profiles that were matched to a FLIP product and the average number of FLIP products that were matched to each FID food profile, were determined. Nutritional composition, specifically calories (kcal), total fat (g), saturated fats (g), trans fats (g), sodium (mg), cholesterol (mg), carbohydrates (g), sugars (g), fibre (g), and protein (g) per 100g for FID food profiles were compared to the nutritional composition of FLIP food profiles using Mann-Whitney U tests because the means of the food profiles at the food category level were not normally distributed. Differences were considered statistically significant at p<0.05. All statistical analyses were conducted using SAS version 9.4 (SAS Institute Inc. Cary, NC).

## Results

### Matching FID file food profiles and FLIP products

Overall, 56.1% (n = 1561) FID food profiles were matched to one or more FLIP products (**Table 2**). Food groups with the greatest proportion of FID food profiles matched to one or more FLIP products were *cereals and other grain products* (81.8%), *bakery products* (80.9%), and *sauces*, *dips*, *gravies*, *and condiments* (80.4%) (Table 2). The food groups with the lowest proportion of FID food profiles matched to one or more FLIP products were *meat*, *poultry*, *their products and substitutes* (32.1%), and *vegetables* (36.4%) (Table 2).

**Table 2:** Number and proportion (%) of FID food profiles that were matched with a FLIP product and the average number and range of FLIP matches per FID food profile, overall and by major food group (n = 2785 FID food profiles) ^a^.

Food Group	n	FID food profiles with a FLIP product match	Average number (range) of FLIP product matches per FID food profile
Cereals and Other Grain Products	143	117 (81.8%)	14.7 (1–162)
Bakery Products	236	191 (80.9%)	13.1 (1–109)
Sauces, Dips, Gravies and Condiments	51	41 (80.4%)	13.0 (1–99)
Sugars and Sweets	136	104 (76.5%)	13.1 (1–157)
Snacks	54	41 (75.9%)	15.8 (1–147)
Nuts and Seeds	78	59 (75.6%)	7.5 (1–28)
Soups	152	113 (74.3%)	4.7 (1–69)
Dairy Products and Alternatives	171	122 (71.3%)	11.3 (1–151)
Fats and Oils	111	77 (69.4%)	8.1 (1–76)
Potatoes	15	10 (66.7%)	12.6 (1–41)
Legumes	93	58 (62.4%)	7.6 (1–66)
Baby and Infant Foods	79	49 (62%)	4.4 (1–25)
Beverages	142	83 (58.5%)	12.3 (1–79)
Desserts	28	15 (53.6%)	14.8 (1–48)
Meal Replacements and Supplements	17	9 (52.9%)	8.7 (1–26)
Combination Dishes	2	1 (50%)	2.0 (2–2)
Miscellaneous Category	63	28 (44.4%)	6.6 (1–49)
Fruit and Fruit Juices	232	101 (43.5%)	7.5 (1–72)
Marine and Freshwater Animals	143	59 (41.3%)	5.9 (1–41)
Vegetables	319	116 (36.4%)	7.6 (1–140)
Meats and Alternatives	520	167 (32.1%)	6.7 (1–59)
**Grand Total**	**2785**	**1561 (56.1%)**	9.7 (1–162)

^a^ Unprocessed meat, fish, poultry, fruit, and vegetables were not matched with data from FLIP database of prepackaged foods, thus, 1,224 (43.9%) FID products could not be matched for this and other reasons (e.g. FID food profiles differed too much from FLIP products).

Abbreviations: FID = Food and Ingredient Details; FLIP = Food Label Information Program.

### Comparison of the nutrient composition of FID and FLIP food profiles

For the majority of food categories and nutrients, there was no statistically significant difference between the underlying FLIP and FID food profile distributions (**Table 3**). The most common nutrients for which there were statistically significant differences between the FLIP and FID food profiles were for saturated fats, fibre, cholesterol, and total fats with differences in nine, seven, six, and four of the 21 food categories, respectively (Table 3). Median saturated fat levels were higher for the FID food profiles in seven of the nine food categories in which there was a statistically significant difference underlying the distributions between the two datasets, with the remaining two food categories having similar median saturated fat values. The difference in the median saturated fat values amongst food categories ranged from 0g to 0.9g per 100g. Median total fat levels were higher in the FID food profiles for three of the four food categories with significant differences in fat levels between the two datasets and no difference in medians for the fourth. Median cholesterol levels were also higher in the FID food profiles for three of the six food categories with significant differences in cholesterol levels between the two datasets and no difference in medians for the other three. Median fibre levels were similar for five of the seven food categories in which there were significant differences in the distribution between the two datasets. For two of the seven categories, *sauces*, *dips*, *gravies*, *and condiments* and *soups* the median difference was 0.7g and 0.2g per 100g, respectively, with both of them higher in the FID food profiles. *Meat*, *poultry*, *their products and substitutes* had the most nutrients with differences between the FID and FLIP food profiles (n = 6) followed by *sauces* (n = 4) and *soups* (n = 3) (Table 3).

**Table 3:** Comparison of nutrient compositions for aggregated FLIP food profiles and FID food profiles by food category (n = 1561).

Food Group	Number of food profiles (n)	Nutrient	Aggregated FLIP food profiles				FID food profiles				p-value^a^
**Mean (SD)**	**Min**	**50th (25th, 75th)**	**Max**	**Mean (SD)**	**Min**	**50th (25th, 75th)**	**Max**
**Bakery Products**	**191**	**Calories (kcal)**	371.7 (91.2)	200	379 (281.8, 442)	592	371 (86.6)	201	389 (287, 436)	558	0.9664
		**Fat (g)**	11.5 (8.3)	0	9.9 (3.9, 18)	32	11.3 (8.3)	0.3	9.8 (3.5, 18.3)	38.5	0.5951
		**Saturated Fat (g)**	3.4 (4)	0	1.8 (0.6, 5)	21.6	3.2 (3.4)	0	1.7 (0.8, 4.9)	20.1	0.4717
		**Cholesterol (mg)**	6.7 (12.3)	0	0 (0, 7.5)	65.5	6.5 (22.4)	0	0 (0, 1)	221	0.0001
		**Sodium (mg)**	468.3 (282.2)	0	413.9 (299.3, 561.2)	2838.3	511.1 (357.3)	0	459 (344, 593)	4029.7	0.1154
		**Carbohydrates (g)**	59.5 (12)	33.6	60.5 (49.1, 68.9)	90.7	59.8 (11.4)	35.5	61 (49.2, 68.9)	86.1	0.7718
		**Protein (g)**	8.6 (3.5)	2.1	8.6 (6.2, 10.4)	29.4	8.6 (3.7)	2.6	8.8 (6.1, 10.6)	31.4	0.9402
		**Sugars (g)**	14.5 (14.5)	0	6.7 (3.2, 26.9)	64.1	15.1 (14.9)	0	7.3 (3.5, 26.4)	61.3	0.7059
		**Fibre (g)**	4.4 (3.1)	0	3.8 (2.3, 5.8)	17.7	4.5 (3.2)	0.4	3.3 (2.2, 6.3)	20.9	0.7373
**Beverages**	**83**	**Calories (kcal)**	85 (118.6)	0	39.1 (20.4, 91)	428.6	101 (137.8)	0	40.5 (18, 112)	460	0.6054
		**Fat (g)**	0.6 (2.2)	0	0 (0, 0)	12.9	0.6 (1.9)	0	0 (0, 0.1)	15.9	0.001
		**Saturated Fat (g)**	0.4 (1.6)	0	0 (0, 0)	10	0.3 (0.7)	0	0 (0, 0)	4.9	< .0001
		**Cholesterol (mg)**	0.8 (3.6)	0	0 (0, 0)	28.6	0.6 (2.8)	0	0 (0, 0)	20	0.1582
		**Sodium (mg)**	50 (129.3)	0	8 (2.7, 26.6)	769.2	46.5 (105)	0	10 (4, 35)	594	0.4375
		**Carbohydrates (g)**	19.6 (27.8)	0	9.8 (5.1, 13.4)	100	23.3 (31.3)	0	10.4 (3.5, 17.8)	98.9	0.4723
		**Protein (g)**	0.8 (2)	0	0 (0, 0.1)	8.6	0.9 (2.4)	0	0 (0, 0.2)	12.2	0.806
		**Sugars (g)**	15.1 (21.6)	0	8 (0.5, 12.6)	90.6	17.3 (26.7)	0	8.5 (0, 13.3)	95.3	0.9575
		**Fibre (g)**	1.1 (7.5)	0	0 (0, 0)	66.7	0.5 (2.7)	0	0 (0, 0.1)	23.3	0.8969
**Cereals and Other Grain Products**	**117**	**Calories (kcal)**	279 (120.7)	37	348.2 (140.6, 368.3)	444.8	280.3 (129.3)	25	358 (130, 379)	475	0.3318
		**Fat (g)**	2.4 (2.7)	0	1.4 (0.6, 3.9)	16.2	2.8 (3.4)	0	1.5 (0.6, 3.9)	19.6	0.5655
		**Saturated Fat (g)**	0.5 (0.8)	0	0.2 (0, 0.5)	5.3	0.6 (1.3)	0	0.3 (0.1, 0.6)	10.8	0.0013
		**Cholesterol (mg)**	1.2 (6.3)	0	0 (0, 0)	47	1.7 (9.5)	0	0 (0, 0)	84	0.41
		**Sodium (mg)**	138.5 (235.4)	0	19.2 (2.4, 194.5)	1688.2	155.5 (302.7)	0	7.5 (3, 171.5)	2152	0.7794
		**Carbohydrates (g)**	57 (25.8)	7.7	69 (28.7, 78.2)	92.6	56.6 (26.9)	5	70 (26.8, 77.8)	91.3	0.8925
		**Protein (g)**	8.9 (8.7)	0	7.3 (4.6, 11.5)	76.7	9 (8.7)	0	7.9 (4, 12.5)	75.2	0.8468
		**Sugars (g)**	5.9 (10)	0	1 (0, 6.6)	54.8	6.4 (10.7)	0	0.9 (0.2, 6.4)	52.2	0.13
		**Fibre (g)**	6 (6.3)	0	3.5 (1.8, 9.3)	36.7	5.9 (6.7)	0.1	3.3 (1.8, 8.2)	42.7	0.9094
**Combination Dishes** ^**b**^	**1**	**Calories (kcal)**	104.3 (.)	104.3	104.3 (104.3, 104.3)	104.3	106.6 (.)	106.6	106.6 (106.6, 106.6)	106.6	.
		**Fat (g)**	2.1 (.)	2.1	2.1 (2.1, 2.1)	2.1	1.2 (.)	1.2	1.2 (1.2, 1.2)	1.2	.
		**Saturated Fat (g)**	0.2 (.)	0.2	0.2 (0.2, 0.2)	0.2	0.2 (.)	0.2	0.2 (0.2, 0.2)	0.2	.
		**Cholesterol (mg)**	0 (.)	0	0 (0, 0)	0	0.9 (.)	0.9	0.9 (0.9, 0.9)	0.9	.
		**Sodium (mg)**	248.7 (.)	248.7	248.7 (248.7, 248.7)	248.7	198.2 (.)	198.2	198.2 (198.2, 198.2)	198.2	.
		**Carbohydrates (g)**	18.3 (.)	18.3	18.3 (18.3, 18.3)	18.3	19.9 (.)	19.9	19.9 (19.9, 19.9)	19.9	.
		**Protein (g)**	3.2 (.)	3.2	3.2 (3.2, 3.2)	3.2	3.7 (.)	3.7	3.7 (3.7, 3.7)	3.7	.
		**Sugars (g)**	2.2 (.)	2.2	2.2 (2.2, 2.2)	2.2	2.9 (.)	2.9	2.9 (2.9, 2.9)	2.9	.
		**Fibre (g)**	0.7 (.)	0.7	0.7 (0.7, 0.7)	0.7	1.8 (.)	1.8	1.8 (1.8, 1.8)	1.8	.
**Dairy Products and Alternatives**	**122**	**Calories (kcal)**	172.1 (121.2)	21.2	112.2 (66.8, 290.4)	404.5	166.7 (123.2)	19	97.5 (71, 266)	452	0.5628
		**Fat (g)**	10.6 (10.8)	0	4.9 (1.5, 19.1)	34.6	10.3 (11.1)	0	3.9 (1.4, 18.3)	35.6	0.7848
		**Saturated Fat (g)**	6.5 (6.9)	0	2.8 (0.9, 12.4)	21.5	6.3 (7)	0	2.1 (0.6, 11.3)	24.6	0.6633
		**Cholesterol (mg)**	33.8 (34.6)	0	16.1 (5.4, 63.4)	109.4	33.5 (36)	0	13 (5.7, 64)	128.5	0.919
		**Sodium (mg)**	362.4 (485.8)	19.7	68.7 (45.7, 617.4)	2279.2	386.1 (541.4)	4	66 (43, 615)	1951	0.7461
		**Carbohydrates (g)**	8.4 (7.7)	0	6.4 (3.6, 11.9)	56.8	8.3 (7.6)	0	5.9 (3.4, 12.3)	54.4	0.7772
		**Protein (g)**	10.8 (9.8)	0.3	6.2 (3.5, 18.6)	43.8	10.4 (9.3)	0	4.9 (3.3, 19.7)	35.8	0.6326
		**Sugars (g)**	5.9 (7.5)	0	4.5 (1.2, 9.3)	56.2	6.1 (7.5)	0	4.3 (1.1, 9.6)	54.4	0.5724
		**Fibre (g)**	0.2 (0.5)	0	0 (0, 0.1)	3.4	0.1 (0.3)	0	0 (0, 0)	2	0.0022
**Desserts**	**15**	**Calories (kcal)**	263.7 (115.5)	120.4	242.4 (160, 307.3)	528.6	217.4 (65.6)	127	216 (181, 249)	358	0.34
		**Fat (g)**	12.4 (9.4)	2.1	10.2 (5.1, 15.9)	33.8	10.5 (7.4)	1.5	8.6 (3.6, 16.2)	25.3	0.7088
		**Saturated Fat (g)**	7.6 (5.8)	1.3	6.2 (3.2, 10.2)	20.4	6 (5.1)	0.8	4.4 (2.3, 10.3)	17.9	0.4306
		**Cholesterol (mg)**	34 (27.2)	7.3	29.1 (13.5, 48.6)	110.9	28.5 (23)	6	27 (13, 34)	92	0.4066
		**Sodium (mg)**	115.8 (59.4)	66	94.2 (77.6, 125.5)	272.7	84.9 (33.9)	48	74 (63, 92)	162	0.0251
		**Carbohydrates (g)**	35 (10.5)	21.1	31.4 (25.7, 47.4)	49.8	28.4 (7.1)	19.8	25.7 (22.2, 37.1)	39.6	0.068
		**Protein (g)**	3.8 (1.1)	2.2	3.8 (2.9, 4.4)	6.8	3.8 (0.8)	2.1	3.8 (3.2, 4.4)	5	0.6782
		**Sugars (g)**	25.1 (8.2)	8.7	24.6 (20.3, 31.3)	40.8	19.7 (4.4)	6.4	20.7 (18.3, 22.1)	25.4	0.0225
		**Fibre (g)**	1.1 (0.8)	0	1.3 (0.2, 1.7)	2.3	1.4 (1.9)	0	0.8 (0, 1.2)	6.6	0.5882
**Fats and Oils**	**77**	**Calories (kcal)**	521.3 (293.2)	40.4	406.5 (300, 862.1)	1009.3	535.2 (289.9)	47	499 (240, 883)	902	0.6579
		**Fat (g)**	54.9 (36.4)	0	42.1 (25.8, 97.3)	108.7	56.2 (36.5)	0.2	55.1 (20, 100)	100	0.5846
		**Saturated Fat (g)**	11.6 (14.9)	0	5.1 (2.5, 15)	82	12.4 (15.5)	0.1	7.7 (3, 13.8)	86.5	0.4544
		**Cholesterol (mg)**	21.5 (45.7)	0	0 (0, 28)	209.4	22.1 (49.6)	0	0 (0, 24)	256	0.9218
		**Sodium (mg)**	547.5 (636)	0	643.8 (0, 770.8)	5020	581.1 (511.8)	0	643 (1, 901)	2684	0.2128
		**Carbohydrates (g)**	7.2 (8.6)	0	3 (0, 15.1)	32.3	8.7 (11.1)	0	2.5 (0, 15.6)	40	0.4249
		**Protein (g)**	0.7 (1.5)	0	0.2 (0, 0.9)	12	0.7 (0.9)	0	0.3 (0, 1)	5.1	0.2674
		**Sugars (g)**	4.8 (7.1)	0	0 (0, 6.7)	32.3	5.2 (7.9)	0	0.1 (0, 8.8)	38.7	0.6308
		**Fibre (g)**	0 (0.2)	0	0 (0, 0)	1.5	0.2 (0.4)	0	0 (0, 0.1)	1.9	< .0001
**Baby and Infant Foods**	**49**	**Calories (kcal)**	184.9 (161.1)	29.7	87.5 (61.4, 384.1)	500	188.1 (157.3)	24	118 (58, 380)	500	0.8842
		**Fat (g)**	3.2 (4.8)	0	1.6 (0.3, 4.5)	28.6	3.1 (4.1)	0	1.9 (0.1, 3.7)	21.4	0.949
		**Saturated Fat (g)**	0 (0.2)	0	0 (0, 0)	1	0.9 (1.1)	0	0.5 (0, 1.2)	4.5	< .0001
		**Cholesterol (mg)**	0 (0)	0	0 (0, 0)	0	2.6 (2.9)	0	1.6 (0, 5)	10.4	< .0001
		**Sodium (mg)**	59.8 (79.1)	0	25.8 (9.4, 80)	392.9	59.1 (96.6)	0	26 (3, 72)	529	0.3704
		**Carbohydrates (g)**	33.9 (29.1)	7.3	16 (12.1, 67.3)	90.1	35.4 (28.8)	5.3	21.3 (11.8, 71.5)	90	0.6164
		**Protein (g)**	5.3 (4.9)	0	3.4 (1.5, 7.3)	17.5	5.5 (5.2)	0	3.7 (1.2, 10)	18.1	0.983
		**Sugars (g)**	11.5 (12.5)	0	7 (3.6, 13.6)	57.1	14.7 (14.2)	0	10.2 (3.3, 20.9)	57.2	0.2849
		**Fibre (g)**	2 (1.9)	0	1.6 (1.1, 2.3)	9.5	3.2 (3.3)	0	1.7 (1.4, 3.5)	14.3	0.0759
**Fruit and Fruit Juices**	**101**	**Calories (kcal)**	112.2 (102.2)	0	58.6 (43.3, 154.6)	352.1	104.8 (97.1)	21	57 (46, 115)	346	0.7243
		**Fat (g)**	0.7 (3.3)	0	0 (0, 0)	21.8	0.7 (2.4)	0	0.1 (0.1, 0.3)	15.3	< .0001
		**Saturated Fat (g)**	0.1 (0.5)	0	0 (0, 0)	3.6	0.1 (0.3)	0	0 (0, 0)	2.1	< .0001
		**Cholesterol (mg)**	0 (0.1)	0	0 (0, 0)	1.3	0 (0)	0	0 (0, 0)	0	0.3246
		**Sodium (mg)**	67.1 (294)	0	4.9 (1.4, 9.8)	1857.1	38.3 (183.9)	0	4 (2, 8)	1556	0.7113
		**Carbohydrates (g)**	25.9 (24.9)	0	13.7 (10.2, 33.9)	85	25.9 (25.5)	3.8	13.8 (10.3, 27.6)	88.3	0.9099
		**Protein (g)**	0.8 (0.9)	0	0.5 (0.2, 1)	5	0.8 (0.9)	0	0.5 (0.3, 0.9)	4.1	0.2702
		**Sugars (g)**	18.9 (18.5)	0	11.2 (8.1, 21.4)	77.1	19.6 (19.4)	0	12.3 (8.8, 20.3)	80.7	0.6748
		**Fibre (g)**	2.1 (2.9)	0	1 (0.3, 3.1)	15	2.1 (2.5)	0	1 (0.4, 2.6)	9.9	0.3427
**Legumes**	**58**	**Calories (kcal)**	161.4 (106.5)	15.7	122.7 (91.1, 197.5)	500	183.9 (111.8)	22	137.5 (112, 290)	476	0.2061
		**Fat (g)**	2.8 (5.4)	0	1 (0.4, 3.2)	35.7	4 (6.6)	0.2	1 (0.5, 4.2)	29.5	0.4378
		**Saturated Fat (g)**	0.6 (2.5)	0	0.1 (0, 0.5)	19.3	0.6 (1)	0	0.2 (0.1, 0.6)	4.6	0.036
		**Cholesterol (mg)**	0.8 (2.7)	0	0 (0, 0)	16.5	0.5 (2.5)	0	0 (0, 0)	17	0.0499
		**Sodium (mg)**	187.9 (244.7)	0.9	71.8 (4.4, 305.6)	958.5	253.4 (348.7)	0	230.5 (6, 400)	1770	0.4265
		**Carbohydrates (g)**	24.3 (18.6)	2.9	18.1 (12.8, 24.4)	63	25.7 (19.9)	1.7	20.8 (9.8, 26.2)	63.7	0.5308
		**Protein (g)**	10.8 (7.4)	0.9	7.8 (5.5, 17.3)	24.8	12.6 (8)	1.1	8.9 (7.5, 21.3)	32	0.1338
		**Sugars (g)**	2.1 (2.3)	0	1.5 (0.6, 2.7)	14.3	2.4 (2.5)	0	1.8 (0.6, 3.3)	10.7	0.6683
		**Fibre (g)**	7.1 (6)	0	4.7 (3.1, 8)	21.8	7.2 (4.9)	0	6.1 (4.3, 9)	24.7	0.273
**Marine and Freshwater Animals**	**59**	**Calories (kcal)**	133.1 (53.5)	57.9	123.5 (89.8, 167.1)	356.3	138.1 (54.2)	51	129 (91, 172)	290	0.5775
		**Fat (g)**	5.3 (5.6)	0.4	3.3 (0.9, 8.8)	32.5	5 (4.6)	0.4	3.4 (1.4, 7.9)	19.6	0.9143
		**Saturated Fat (g)**	1.1 (1.1)	0	0.7 (0.2, 1.8)	5.5	1 (0.9)	0	0.9 (0.2, 1.5)	4.1	0.6766
		**Cholesterol (mg)**	70.3 (53.1)	8.7	57.1 (44.8, 77.3)	320.9	80.1 (81.7)	0	57 (47, 79)	479	0.8611
		**Sodium (mg)**	454.3 (917.5)	0	275 (80.2, 445.2)	5949.2	475.8 (1033)	0	166 (67.9, 414)	7027	0.5997
		**Carbohydrates (g)**	1.9 (4)	0	0 (0, 1.7)	16.2	1.2 (2.8)	0	0 (0, 0.9)	15	0.1452
		**Protein (g)**	19.4 (4.9)	5	20 (16.3, 22.9)	28.7	20.9 (7.7)	5.7	20.4 (17.2, 24.4)	62.8	0.3326
		**Sugars (g)**	0.8 (2.8)	0	0 (0, 0)	16	0.3 (1.3)	0	0 (0, 0)	7.7	0.0862
		**Fibre (g)**	0 (0.1)	0	0 (0, 0)	0.8	0 (0.1)	0	0 (0, 0)	0.5	0.0479
**Meal Replacements and Supplements**	**9**	**Calories (kcal)**	223 (157.1)	59.2	145.9 (84.5, 380.1)	400.7	214.9 (152.4)	37	142 (95, 362)	399	0.791
		**Fat (g)**	4.3 (4.2)	1.1	2.5 (2.3, 5.1)	15	3.8 (3.8)	1.1	2.4 (1.6, 5.2)	12.9	0.6588
		**Saturated Fat (g)**	1.3 (2.2)	0.2	0.5 (0.4, 0.9)	7	1.3 (2.2)	0.2	0.5 (0.3, 0.8)	6.6	0.7363
		**Cholesterol (mg)**	4.3 (5.2)	0	2 (1.4, 3.7)	13.7	5 (4.3)	0	4 (2.3, 4)	12.3	0.2881
		**Sodium (mg)**	201.2 (144.9)	51.8	102.4 (88.8, 324.7)	400	236.9 (178.4)	71	112 (90.7, 385)	505	0.5962
		**Carbohydrates (g)**	36.3 (30.8)	6.7	19.6 (12, 65.1)	80	35.4 (30.1)	2.1	17.8 (13, 66.2)	79.1	1
		**Protein (g)**	11.4 (7.9)	4.2	6.5 (5.7, 17.5)	24.6	11.2 (7.9)	3.9	6.3 (5.5, 19.9)	22.4	0.7239
		**Sugars (g)**	17.1 (20.3)	0.2	4.9 (2.1, 21.2)	52.5	18 (24.8)	0	8.7 (1.6, 23)	65.8	0.953
		**Fibre (g)**	3.3 (3.2)	0.1	1.2 (0.4, 6.3)	7.5	1.8 (2.2)	0	1.2 (0.4, 2.2)	6.7	0.4702
**Meats and Alternatives**	**167**	**Calories (kcal)**	212.3 (139.4)	47.6	190.6 (140, 257.9)	1269.8	211.4 (80.3)	48	204 (154, 254)	541	0.2379
		**Fat (g)**	13.6 (14.4)	0	10.7 (4.6, 19)	117.9	12 (8.2)	0	10.6 (5.5, 16.2)	41.8	0.7878
		**Saturated Fat (g)**	4.8 (5.3)	0	3.3 (1.7, 7.2)	43.2	4.4 (3)	0	3.8 (1.9, 6.5)	13.7	0.797
		**Cholesterol (mg)**	76.6 (55.7)	0	66.8 (54.1, 83.1)	422.4	90.8 (102.6)	0	75.3 (60.2, 86)	884	0.0131
		**Sodium (mg)**	613.6 (443.9)	40	621 (262.1, 841)	2338.9	543 (553.3)	1.2	146 (67, 1038)	2560	0.0282
		**Carbohydrates (g)**	2.9 (5.9)	0	1.8 (0, 3.6)	66.7	2.6 (4.3)	0	0.2 (0, 4.1)	27	0.0114
		**Protein (g)**	19.4 (5.9)	0	18.9 (15.7, 22.8)	47.6	21.7 (6.9)	8.6	20.9 (16.4, 27.2)	55.5	0.0015
		**Sugars (g)**	0.8 (1.1)	0	0.1 (0, 1.3)	6	0.6 (1.9)	0	0 (0, 0.7)	21.8	0.0052
		**Fibre (g)**	0.1 (0.3)	0	0 (0, 0.2)	2	0.1 (0.3)	0	0 (0, 0)	2.3	< .0001
**Miscellaneous Category**	**28**	**Calories (kcal)**	178.2 (149.4)	0	164.9 (41, 280.6)	467.4	214.8 (141.9)	0	230.5 (79.5, 323.5)	545	0.3128
		**Fat (g)**	3.9 (6.7)	0	0 (0, 6.8)	27	5.9 (8)	0	3.8 (0.1, 8.8)	35.5	0.0759
		**Saturated Fat (g)**	2 (5.2)	0	0 (0, 0.3)	23.4	2.7 (6.4)	0	0.9 (0, 2.6)	32.5	0.0223
		**Cholesterol (mg)**	0.1 (0.4)	0	0 (0, 0)	2.2	0 (0)	0	0 (0, 0)	0	0.3349
		**Sodium (mg)**	3932 (8746.7)	0	162.6 (32.3, 3455.6)	39000	3130.8 (8830.1)	2	63.5 (25, 436)	38758	0.363
		**Carbohydrates (g)**	27.9 (26.9)	0	27.2 (0, 43.6)	83.3	36.5 (25.3)	0	42 (11.3, 57.3)	73.4	0.152
		**Protein (g)**	12 (16.1)	0	5.6 (0.7, 20)	55.6	13 (18.2)	0	6.9 (0.8, 18.9)	85.6	0.7991
		**Sugars (g)**	7.2 (10.8)	0	0 (0, 10.1)	33.3	9.2 (17.3)	0	2.8 (0.4, 9.1)	73.4	0.2338
		**Fibre (g)**	6.5 (12.4)	0	0 (0, 6.3)	40	12.2 (15.2)	0	6.6 (0, 26.9)	53.2	0.0357
**Nuts and Seeds**	**59**	**Calories (kcal)**	601.6 (85.9)	160	618.2 (566.7, 645)	775.1	584.2 (72.6)	245	587 (570, 608)	718	0.0271
		**Fat (g)**	49.4 (12.5)	0.8	50 (44.5, 54.7)	76.5	50.4 (12.7)	2.2	49.9 (47, 55.2)	76.1	0.5979
		**Saturated Fat (g)**	8.2 (7.2)	0	6.7 (4.7, 9.2)	46.4	8.2 (8.2)	0.4	6.7 (4.5, 8.7)	57.2	0.7549
		**Cholesterol (mg)**	0.2 (1.7)	0	0 (0, 0)	13.3	0 (0)	0	0 (0, 0)	0	0.3256
		**Sodium (mg)**	119.6 (170.2)	0	10 (2, 263.5)	524.6	126.9 (188.8)	0	13 (4, 273)	655	0.4489
		**Carbohydrates (g)**	24.8 (11.5)	7.6	21.9 (18, 30)	66	23.8 (9.8)	8.1	21.6 (17.6, 28.7)	53	0.7631
		**Protein (g)**	18.5 (6.6)	4	18.8 (15.9, 21.5)	34	18.7 (7.2)	2.9	19.3 (15.3, 21.4)	39.5	0.9399
		**Sugars (g)**	7.8 (9.9)	0	4.5 (3.3, 7.2)	54	6.8 (8.4)	0.5	4.6 (4, 6.6)	43.2	1
		**Fibre (g)**	8.7 (5.3)	2.3	8 (6, 10)	36	8.7 (5.3)	2	8.2 (5.6, 9.9)	34.4	0.9413
**Potatoes**	**10**	**Calories (kcal)**	176 (115.9)	47.4	165.1 (69.6, 230.6)	368.5	172.7 (98.1)	77	152.5 (87, 219)	354	0.9097
		**Fat (g)**	3.5 (3.8)	0	1.9 (0, 6.9)	10.1	3.5 (4.2)	0.1	2.1 (0.1, 5.5)	11.6	0.8498
		**Saturated Fat (g)**	0.5 (0.5)	0	0.5 (0, 0.8)	1.6	0.7 (0.8)	0	0.5 (0, 1)	2.3	0.4713
		**Cholesterol (mg)**	0.2 (0.6)	0	0 (0, 0)	2.1	0 (0)	0	0 (0, 0)	0	0.1681
		**Sodium (mg)**	400.2 (527)	8	243.7 (89, 347.2)	1740.6	335.3 (641.7)	4	59.5 (5, 332)	2095	0.3445
		**Carbohydrates (g)**	33 (24.5)	11	24.2 (15.8, 33.5)	78.1	34.1 (23.3)	17.5	25.2 (20.1, 28.5)	81.2	0.6776
		**Protein (g)**	3.3 (2.3)	1.2	2.6 (1.8, 3.2)	7.9	3.5 (2.7)	1.7	2.2 (2, 2.8)	8.9	1
		**Sugars (g)**	1.7 (1.7)	0	1.4 (0.1, 3.5)	4.4	1.1 (1)	0.3	0.8 (0.3, 1.3)	3.4	0.6891
		**Fibre (g)**	3 (1.8)	0.9	2.4 (2.2, 2.9)	6.6	2.8 (1.6)	1.4	2.2 (1.6, 3.3)	6.5	0.5708
**Sauces, Dips, Gravies and Condiments**	**41**	**Calories (kcal)**	160 (118.2)	28	129 (64.6, 217.7)	486.3	159.5 (142.8)	11	95 (53, 220)	535	0.5906
		**Fat (g)**	4 (9.1)	0	0.4 (0, 3.3)	49.3	6.1 (10)	0	2.1 (0.3, 8)	46.1	0.029
		**Saturated Fat (g)**	1.2 (2.6)	0	0 (0, 0.6)	11.7	2.4 (5.3)	0	0.3 (0, 2.4)	26.4	0.0084
		**Cholesterol (mg)**	3.9 (14.9)	0	0 (0, 0)	86.7	14.1 (64.7)	0	0 (0, 3)	413.5	0.0101
		**Sodium (mg)**	2603.2 (2764.3)	18.4	1509.4 (572.3, 4532)	13428.6	2543.2 (2673.4)	13	1307 (570, 4152)	11588	0.8239
		**Carbohydrates (g)**	25.6 (21.1)	3	19.8 (8.1, 36.4)	70.4	22.3 (21.6)	1	10.9 (5.3, 38.2)	65.1	0.1214
		**Protein (g)**	4.2 (4.4)	0.2	2.1 (1.2, 5.7)	17.8	4.8 (4.2)	0	3.7 (1.3, 8.8)	15.3	0.5283
		**Sugars (g)**	10.7 (10.6)	0	6.3 (2.3, 17.2)	42	7.6 (9.4)	0	3.3 (0.5, 11.8)	33.2	0.1318
		**Fibre (g)**	0.3 (1.1)	0	0 (0, 0.1)	6.4	1.3 (1.4)	0	0.7 (0.3, 2)	5.9	< .0001
**Snacks**	**41**	**Calories (kcal)**	471.3 (73.1)	250	485 (455.8, 520)	566.6	478.7 (61.2)	345	484 (431, 531)	583	0.8383
		**Fat (g)**	22.4 (10.1)	1.6	24.3 (19.1, 29.6)	38	23 (11.9)	2.4	25.6 (15.6, 33.4)	43.6	0.6296
		**Saturated Fat (g)**	5.4 (5.6)	0	3.6 (2.5, 6.3)	27.6	5.7 (5.8)	0.4	3.9 (2.2, 7.2)	29	0.8601
		**Cholesterol (mg)**	7.8 (22.9)	0	0 (0, 0.9)	91.6	6.6 (23.5)	0	0 (0, 0)	111	0.0652
		**Sodium (mg)**	547.1 (380.7)	0	545.8 (245, 695)	1473.4	563.7 (505)	6	488 (202, 691)	2081	0.6231
		**Carbohydrates (g)**	57.7 (15.4)	4.6	61.9 (53, 67.2)	78.3	59 (19)	0	60.8 (53.3, 71.4)	83.4	0.5779
		**Protein (g)**	10.2 (9.1)	2.2	7.8 (6.1, 9.8)	46.4	10.7 (10.7)	2.3	7.5 (6.3, 10)	61.3	0.9704
		**Sugars (g)**	8.8 (12.2)	0	3.5 (1, 10.1)	39.7	7.1 (13.6)	0	0.9 (0.6, 4.8)	53.2	0.1082
		**Fibre (g)**	5.2 (3.3)	0	4.7 (3.2, 6.2)	14	5.6 (3.9)	0	4.2 (3.4, 6.9)	17.7	0.9224
**Soups**	**113**	**Calories (kcal)**	90.5 (114.7)	0	49.3 (31.4, 76.2)	460	88.5 (115.6)	3	47 (30, 74)	446	0.7432
		**Fat (g)**	2.2 (3.4)	0	1 (0.3, 2.8)	23	2.8 (4.2)	0	1.2 (0.5, 3.3)	24.1	0.1698
		**Saturated Fat (g)**	0.6 (1.5)	0	0 (0, 0.6)	9	0.9 (1.8)	0	0.4 (0.1, 1.1)	13.8	< .0001
		**Cholesterol (mg)**	2.6 (5.6)	0	0.9 (0, 3.8)	47.6	3.8 (7.8)	0	2 (0, 4)	74	0.0187
		**Sodium (mg)**	1034.5 (2448.4)	7.8	277.2 (228.9, 491.3)	16678.9	1536 (4174.6)	27	324.9 (242, 563)	26000	0.1407
		**Carbohydrates (g)**	14.5 (21.2)	0.2	6.8 (4.4, 9.4)	76	12.5 (18.2)	0	6.3 (3.7, 9)	73.7	0.3761
		**Protein (g)**	3 (3.9)	0	1.5 (1, 2.7)	23.3	3.5 (4.9)	0.1	1.6 (0.9, 2.8)	20.5	0.6282
		**Sugars (g)**	2.6 (3.5)	0	1.1 (0.6, 2.7)	15.8	2.2 (3.6)	0	1 (0.4, 2.1)	17.5	0.1956
		**Fibre (g)**	1.1 (2)	0	0.5 (0, 1.1)	14	1.1 (1.3)	0	0.7 (0.3, 1.3)	10	0.0347
**Sugars and Sweets**	**104**	**Calories (kcal)**	323.8 (158.2)	0	336.9 (222.1, 454.5)	583.3	328.3 (141.4)	0	356 (246, 410)	642	0.8828
		**Fat (g)**	9.6 (13.2)	0	1.8 (0, 18.5)	52.4	8.6 (11.7)	0	2.1 (0.1, 16.2)	52.3	0.8324
		**Saturated Fat (g)**	5 (7.5)	0	0.8 (0, 7.1)	30.4	4.6 (7.3)	0	1 (0, 5.2)	32.3	0.9468
		**Cholesterol (mg)**	5.2 (9.1)	0	0 (0, 7.8)	47.6	6 (26.3)	0	0 (0, 3)	258	0.1303
		**Sodium (mg)**	255 (739.1)	0	69.9 (24.6, 137.5)	4400	205.3 (507.5)	0	72 (28, 168)	3750	0.6558
		**Carbohydrates (g)**	59 (25.6)	0.5	61.3 (48.1, 77.8)	117.2	63.8 (25.2)	4.2	67.4 (54.5, 81.7)	100	0.0791
		**Protein (g)**	3.2 (5)	0	1.1 (0.2, 5.2)	39.5	2.9 (3.6)	0	1.8 (0.1, 4.6)	15.7	0.9935
		**Sugars (g)**	44.9 (24.6)	0	46.8 (27.8, 60.4)	112.4	48.2 (26.2)	0	50.2 (24.3, 67.1)	99.8	0.236
		**Fibre (g)**	1.5 (2.4)	0	0.4 (0, 2.3)	10.7	1.4 (2.3)	0	0.3 (0, 2.5)	16.6	0.6747
**Vegetables**	**116**	**Calories (kcal)**	74.3 (81.9)	0	41.9 (28.4, 87.8)	485.7	58.3 (58.6)	11	37.5 (24, 72)	324	0.0862
		**Fat (g)**	1.3 (4.1)	0	0 (0, 0.5)	29.3	0.7 (1.6)	0.1	0.3 (0.2, 0.5)	14.1	< .0001
		**Saturated Fat (g)**	0.1 (0.4)	0	0 (0, 0)	3.2	0.1 (0.2)	0	0.1 (0, 0.1)	1.9	< .0001
		**Cholesterol (mg)**	0.1 (0.5)	0	0 (0, 0)	4	0 (0.2)	0	0 (0, 0)	2	0.101
		**Sodium (mg)**	264.6 (482.2)	0	68.1 (18.1, 250.4)	2625	178.9 (320.2)	0.2	45.5 (12.5, 244.5)	1674	0.1621
		**Carbohydrates (g)**	11.9 (10.8)	0	7.5 (5, 16)	62.5	12 (13.1)	1.9	7.8 (4.7, 14.7)	75.4	0.679
		**Protein (g)**	3.2 (5)	0	2 (1.2, 3)	31.4	2.9 (3.9)	0.3	1.9 (1, 3.1)	36.2	0.7647
		**Sugars (g)**	4.6 (6.3)	0	2.4 (1, 4.6)	27.5	4.2 (6.6)	0.3	2.5 (1.4, 3.9)	41.1	0.6719
		**Fibre (g)**	3 (4.1)	0	2 (1.4, 3)	28.6	3.2 (6.9)	0.4	2 (1.5, 2.9)	70.1	0.8418

^a^ P-value determined using Mann Whitney U tests.

^b^ Unable to determine p-values for the category “Combination Dishes” because there was only one food profile in this category.

Abbreviations: FID = Food and Ingredient Details; FLIP = Food Label Information Program; Kcal = calories; SD = Standard Deviation

## Discussion

This study was undertaken to compare the nutritional composition of the food profiles in the CCHS 2015 *Food and Ingredient Details* file to products in a large representative database of prepackaged food and beverage products available in the Canadian food supply as of 2017. The results demonstrate that although there was fairly high overlap in the types of foods in the FID file and the FLIP database, a proportion of each of the datasets were left unmatched. Secondly, while many food categories had no difference in the nutritional composition of food profiles between FID and FLIP, there were some differences in nutritional composition between composite values compared, which could be both methodologically and nutritionally meaningful in the appraisal of dietary intake data and the establishment of food policies.

Given the misalignment identified in this study between the FID food profiles and a representative sample of Canadian prepackaged foods on the market, the combined use of a branded prepackaged food composition database with CNF data for whole and fresh foods may be a worthwhile consideration. Almost half (43.9%) of the FID food profiles did not have a match to a FLIP product. This is likely due to the fact that the FLIP database is composed of prepackaged foods and beverages. Although there are some fresh foods (e.g. fruits, vegetables, meat) in the FLIP database that are packaged, and therefore were included in the collection, many other fresh foods were not. Whole and fresh foods are not subject to traditional methods of reformulation and rapid change in nutritional content, and therefore, we did not need to compare the nutritional composition of these foods with those previously collected as part of the CNF. Given the nature of the FLIP database, it is unsurprising that the food categories in which products are primarily composed of packaged foods (e.g. *cereals and other grain products*, *bakery products*, *sauces*, *dips*, *gravies*, *and condiments*) had the most FID food profiles with matches.

There were also some FLIP foods that did not get matched to a FID food profile because they were a combination of foods in a package with one combined NFt, or because they were deemed to be too different from any FID food profiles to be matched (e.g. chia pudding, cookie butter, palm fruit in syrup, kale bread, tiger nuts, powdered peanut butter). The former example presents a limitation of this study, in which the researchers were unable to separate the nutrient information for two items when they were packaged together. The latter, even though such foods represented a relatively small proportion of the FLIP database, indicates that national food composition databases may need to be updated more frequently as food preferences change and new items are continually being introduced into the marketplace.

The food category with the most differences in nutritional composition between the two databases was *meat and alternatives*. This is an important category and a top source of several nutrients in the Canadian diet, including calories, sodium, saturated fats, and to a lesser extent, sugars [16]. Additionally, 89.4% and 74.2% of respondents in CCHS 2015 reported consuming meat and alternative products, respectively, each day [17]. Thus, if the nutritional composition of the foods available in the marketplace, and therefore the foods consumed by the Canadian population, were different, the results from this survey may not be as representative of actual intakes. The direction of the nutritional differences does not indicate a systematic trend, with median cholesterol and protein being lower in FLIP while median sodium, carbohydrates, and sugars being higher in FLIP and the median fibre being the same, even though the distributions differed. This finding may indicate a poorer nutritional quality of products in the marketplace as FLIP food profiles tended to be higher in some nutrients of public health concern compared to levels in the FID.

The most recent version of the CNF database, 2015, which was used as the basis for the FID file, was updated with a focus on the major contributors of sodium to the diet [18]. Thus, as expected, sodium was one of the nutrients with the fewest food categories with differences between the FID and FLIP food profiles, with only two categories (i.e. *meats and alternatives*, *desserts*). For both these categories, the median sodium content was higher in the FLIP food profiles than in the FID food profiles. While *desserts* is not a top category contributing to sodium intakes, *meats and alternatives*, particularly processed meats, is a major contributor to sodium intakes in Canada [19]. Based on these results it is possible that the current estimates of Canadian sodium intakes, most recently published by Health Canada in 2017, underestimate sodium consumption in the diet [19]. These results are not surprising as several meat subcategories such as packaged deli meats, canned meats, meatballs, meatloaf, and burgers did not make meaningful reductions, and in a few cases (e.g. marinated meat and poultry, chicken wings), sodium levels increased between 2012 and 2017 [20].

Saturated fats, fibre, cholesterol, and total fats were the nutrients with differences between the FID and FLIP food profiles in the most categories. For all these nutrients, the medians were higher for the FID food profiles than for the FLIP food profiles, wherever the medians differed. This finding indicates that the FID food profiles may be overestimating the contribution of foods and beverages to these nutrients compared to the foods available in the current marketplace. Other nutrients (i.e. calories, sodium, sugars, carbohydrates) had higher median levels in the FLIP food profiles in some categories. Although the FID food profiles may be higher or lower in some nutrients than the FLIP food profiles, it is unknown whether these differences would translate into a nutritionally significant difference in intakes when used as the basis for the CCHS 24-hour recalls. The distribution of nutrients in many food categories were not significantly different between the two databases, however, there could be differences at the level of the subcategory that could impact nutrient intakes. Future efforts can utilize the matching between the FLIP 2017 database with the FID food profiles to examine CCHS 24-hour recalls using nutrient data from a representative sample of Canadian foods and beverages that have been systematically collected. Such a matching could also quantify the magnitude of the variation in intake due to the wide range of nutritional levels seen in comparable foods. For example, fat levels in baking products vary more than 4-fold between the 25^th^ and 75^th^ percentiles.

These results can inform the next round of CNF collections in terms of the areas of the highest priority for updating. This study indicated that some of the areas of the FID, or CNF data that are most inconsistent with a database representative of the current Canadian food supply are the food categories of *meats and alternatives* as well as *soups*, and the nutrients saturated fats, total fats, cholesterol, and fibre.

There are several limitations of this study that should be noted. First, the years of data collection between the FID and FLIP differed. Although the 2015 edition of CNF was used as the basis of the FID file, there was no FLIP collection conducted in that year and thus the 2017 version of FLIP was used. Despite this two-year difference, we do not anticipate that the results would have varied significantly. Secondly, the matching of FID food profiles and FLIP products could be considered a subjective process, however, steps were taken to limit subjectivity where possible including the use of a decision-tree to guide the researchers, exact or default matching were available for approximately 75% of foods. Furthermore, a subset of the matches was validated by a second researcher and consensus was reached for any discrepancies. While the FLIP dataset is representative of a large proportion of the total Canadian grocery channel, we are not able to make assertions related to the market share of individual products, which would increase the accuracy in estimating participants’ nutrient exposure when applied to the CCHS. While some food profiles in the CNF were sampled on the basis of products with high market share (i.e. most popular), this process was only done for certain priority foods and may be several years old. Future iterations of food composition databases used to create aggregate nutrient values for use in dietary surveys would benefit by sales weighting composite values captured from current branded food composition databases.

## Conclusion

Results from CCHS nutrition surveys have broad potential applications as they can influence nutrition and agricultural policy decisions, steer research activities examining nutrient disease relationships, influence the development of standardized nutrient reference amounts, and inform dietary programs and guidance [3]. Thus, it is essential that the food composition database used to code for foods reported in the CCHS 24-hour recalls is representative of the current Canadian food supply. Findings from this study demonstrate that the nutritional composition of the FID food profiles are not different that those in a representative sample of Canadian packaged foods collected in 2017 in many food categories, although, there are still instances where the nutritional composition differs. These results can be used to inform future updates and collections of the CNF database, while also providing insights for interpreting CCHS 2015 nutrient intakes.
